# ﻿An image dataset of cleared, x-rayed, and fossil leaves vetted to plant family for human and machine learning

**DOI:** 10.3897/phytokeys.187.72350

**Published:** 2021-12-16

**Authors:** Peter Wilf, Scott L. Wing, Herbert W. Meyer, Jacob A. Rose, Rohit Saha, Thomas Serre, N. Rubén Cúneo, Michael P. Donovan, Diane M. Erwin, María A. Gandolfo, Erika González-Akre, Fabiany Herrera, Shusheng Hu, Ari Iglesias, Kirk R. Johnson, Talia S. Karim, Xiaoyu Zou

**Affiliations:** 1 Department of Geosciences and Earth and Environmental Systems Institute, Pennsylvania State University, University Park, PA 16802, USA Pennsylvania State University University Park United States of America; 2 Department of Paleobiology, Smithsonian Institution, Washington, DC 20013, USA Department of Paleobiology, Smithsonian Institution Washington, DC United States of America; 3 Florissant Fossil Beds National Monument, National Park Service, Florissant, CO 80816, USA Florissant Fossil Beds National Monument, National Park Service Florissant United States of America; 4 School of Engineering, Brown University, Providence, RI 02912, USA Brown University Providence United States of America; 5 Department of Cognitive, Linguistic and Psychological Sciences, Carney Institute for Brain Science, Brown University, Providence, RI 02912, USA Museo Paleontológico E. Feruglio Trelew Argentina; 6 CONICET-Museo Paleontológico Egidio Feruglio, Trelew 9100, Chubut, Argentina epartment of Paleobotany and Paleoecology, Cleveland Museum of Natural History Cleveland United States of America; 7 Department of Paleobotany and Paleoecology, Cleveland Museum of Natural History, Cleveland, OH 44106, USA University of California-Berkeley Berkeley United States of America; 8 University of California-Berkeley, Museum of Paleontology, Berkeley, CA 94720, USA Cornell University Ithaca United States of America; 9 LH Bailey Hortorium, Plant Biology Section, School of Integrative Plant Science, Cornell University, Ithaca, NY, 14853, USA Smithsonian Conservation Biology Institute, National Zoological Park, Front Royal United States of America; 10 Conservation Ecology Center, Smithsonian Conservation Biology Institute, National Zoological Park, Front Royal, VA, 22630, USA Negaunee Integrative Research Center, Field Museum of Natural History Chicago United States of America; 11 Negaunee Integrative Research Center, Field Museum of Natural History, Chicago, IL, 60605, USA Yale University New Haven United States of America; 12 Division of Paleobotany, Peabody Museum of Natural History, Yale University, New Haven, CT 06520, USA Instituto de Investigaciones en Biodiversidad y Ambiente INIBIOMA, CONICET-UNComa San Carlos de Bariloche Argentina; 13 Instituto de Investigaciones en Biodiversidad y Ambiente INIBIOMA, CONICET-UNComa, San Carlos de Bariloche 8400, Río Negro, Argentina Department of Paleobiology, Smithsonian Institution Washington United States of America; 14 University of Colorado Museum of Natural History, Boulder, CO 80503, USA University of Colorado Museum of Natural History Boulder United States of America

**Keywords:** Angiosperms, cleared leaves, data science, fossil leaves, leaf architecture, paleobotany

## Abstract

Leaves are the most abundant and visible plant organ, both in the modern world and the fossil record. Identifying foliage to the correct plant family based on leaf architecture is a fundamental botanical skill that is also critical for isolated fossil leaves, which often, especially in the Cenozoic, represent extinct genera and species from extant families. Resources focused on leaf identification are remarkably scarce; however, the situation has improved due to the recent proliferation of digitized herbarium material, live-plant identification applications, and online collections of cleared and fossil leaf images. Nevertheless, the need remains for a specialized image dataset for comparative leaf architecture. We address this gap by assembling an open-access database of 30,252 images of vouchered leaf specimens vetted to family level, primarily of angiosperms, including 26,176 images of cleared and x-rayed leaves representing 354 families and 4,076 of fossil leaves from 48 families. The images maintain original resolution, have user-friendly filenames, and are vetted using APG and modern paleobotanical standards. The cleared and x-rayed leaves include the Jack A. Wolfe and Leo J. Hickey contributions to the National Cleared Leaf Collection and a collection of high-resolution scanned x-ray negatives, housed in the Division of Paleobotany, Department of Paleobiology, Smithsonian National Museum of Natural History, Washington D.C.; and the Daniel I. Axelrod Cleared Leaf Collection, housed at the University of California Museum of Paleontology, Berkeley. The fossil images include a sampling of Late Cretaceous to Eocene paleobotanical sites from the Western Hemisphere held at numerous institutions, especially from Florissant Fossil Beds National Monument (late Eocene, Colorado), as well as several other localities from the Late Cretaceous to Eocene of the Western USA and the early Paleogene of Colombia and southern Argentina. The dataset facilitates new research and education opportunities in paleobotany, comparative leaf architecture, systematics, and machine learning.

## ﻿Introduction

General patterns of angiosperm leaf architecture, the shape and venation characters of leaves, are well known for very few of the more than 400 angiosperm families. The development of a standard descriptive terminology (von Ettingshausen 1861; Hickey 1973, 1979; Ellis et al. 2009) has catalyzed increased detail and reproducibility in species descriptions of both living and fossil leaves. However, despite the use of numerous visual examples (e.g., Ellis et al. 2009), publications to date do not inform the reader how to accomplish the fundamental task of identifying leaves that, as for the great majority of leaf fossils, are isolated from the rest of the plant and missing diagnostic information from stipules, leaf organization, and reproductive and other organs.

To build their knowledge of leaf architecture, researchers still rely primarily on “oral tradition” from a dwindling number of knowledgeable colleagues and a handful of survey papers and field guides that emphasize purportedly diagnostic leaf features (Hickey and Wolfe 1975; Gentry 1993; da Ribeiro et al. 1999; Keller 2004). There is significant literature on the leaf architecture and leaf-fossil records of various taxa (among many others, von Ettingshausen 1858; Hill 1982; Jones 1986; Manchester 1987; Todzia and Keating 1991; Gandolfo and Romero 1992; Premoli 1996; Fuller and Hickey 2005; Martínez-Millán and Cevallos-Ferriz 2005; Doyle 2007; Kellner et al. 2012). However, many of the most diverse and ecologically significant groups of angiosperms have virtually no documentation of diagnostic leaf-blade features (e.g., Asteraceae, Rubiaceae), and thus their leaf fossils remain largely unrecognized, though probably hidden in plain sight in museum collections (see Wilf 2008; Wilf et al. 2016). More than half of fossil-leaf species in many older monographs are thought to have been misclassified (see Dilcher 1971), and most of the millions of leaf fossils in the general stratigraphic collections of the world’s museums are not yet identified.

Machine-vision algorithms, as seen in popular applications such as LeafSnap (Kumar et al. 2012), Pl@ntNet (Bonnet et al. 2018), and iNaturalist (Van Horn et al. 2018), are making spectacular breakthroughs in automated species identification of live plants; however, they provide little, if any, feedback about the diagnostic features they detect. Few algorithms have attempted to generalize above the species level (Wilf et al. 2016; Carranza-Rojas et al. 2018), and so far the methods do not work on leaf fossils, which mostly represent extinct species and often extinct genera.

Increasing general knowledge of leaf architecture for both human and machine learners depends on the development of customized, accessible, vetted visual libraries that allow rapid morphological comparisons of a high phylogenetic diversity of extant and fossil leaves. The recent proliferation of digitized plant-image resources comprises an invaluable reference for plant morphology, already including tens of millions of digitized herbarium sheets on portals and aggregator sites such as JStor Global Plants (https://plants.jstor.org), iDigBio (https://www.idigbio.org), RecolNat (https://www.recolnat.org), and many others, as well as servers located at numerous individual herbaria worldwide (e.g., Bakker et al. 2020). However, studying leaf comparative morphology is not simple because leaves only represent part of the visual field of a herbarium sheet and appear, with overlaps, at many different angles and sizes. Computer-vision algorithms that blur text or segment leaves from background or from other plant material are likely to help solve this issue (Hussein et al. 2020, 2021; Weaver et al. 2020; de Lutio et al. 2021). However, many visual distractors remain, and critical details of higher-order venation are often not visible in digitized herbarium sheets. Assessing leaf architecture at family level from digital herbaria also requires examination of extremely large numbers of specimens for all but the most species-poor families. In this regard, JStor Global Plants stands out for prioritizing type specimens collated digitally from across the world’s herbaria, thus allowing rapid surveys of the taxa in a family based on protologue voucher material. Finally, digitization efforts are far more advanced in resource-rich countries, whereas many significant collections are located in developing nations where herbarium digitization is occurring at a slower pace.

Cleared or x-rayed leaves from phylogenetically diverse taxa, selectively sampled from vouchered herbarium sheets, remain the most valuable visual reference for comparative study of leaf architecture because they have a similar visual presentation, with high capture of venation detail and comparatively few distractors. Existing collections of this type are fragile, mostly made decades ago as references for fossil leaf identification by selecting leaves from herbarium sheets, then either chemically clearing the specimens of most tissues other than veins and mounting them on glass slides or x-ray imaging them, in either case with extreme care and effort. Most cleared-leaf collections suffer from deterioration of the mounting media, which obscures large areas of the leaves; thus, photographic archiving offers a form of visual preservation before further degradation occurs. The largest and best-known cleared-leaf collections are those of the late Drs. Jack A. Wolfe and Leo J. Hickey, together now forming the National Cleared Leaf Collection (NCLC; NCLC-W and NCLC-H, respectively), housed in the Division of Paleobotany of the Smithsonian Institution National Museum of Natural History (NMNH, repository acronym USNM, Washington, D.C.).

For the many users who may find it challenging to visit these collections in person for suitable lengths of time, many cleared and x-rayed leaf collections are already accessible from various websites or in print. These valuable resources include the NCLC-W and other collections in the Cleared Leaf Image Database (http://clearedleavesdb.org; Das et al. 2014); the NCLC-H served from the Yale Peabody Museum (https://collections.peabody.yale.edu/pb/nclc); the Daniel I. Axelrod cleared-leaf collections of the University of California Museum of Paleontology (UCMP; https://UCMP.berkeley.edu/collections/paleobotany-collection/UCMP-cleared-leaf-collection); the National Museum of Nature and Science (NMNS, Ibaraki, Japan) Cleared Leaf Database by Drs. Toshimasa Tanai and Kazuhiko Uemura (https://www.kahaku.go.jp/research/db/geology-paleontology/cleared_leaf/database/?lg=en); leaf x-ray images of Australian rainforest plants by the late Dr. David C. Christophel and colleagues (Christophel and Hyland 1993; Christophel and Rowett 1996), some of which are maintained in the online Australian Tropical Rainforest Plants identification system (https://apps.lucidcentral.org/rainforest/text/intro/index.html); and the late Dr. Edward P. Klucking’s book series illustrating cleared leaves from selected families (Klucking 1986–2003). We also note an open-access image dataset of cleared leaves from Borneo, consisting of small (1 cm^2^) lamina samples (Blonder et al. 2019; Xu et al. 2021). In most of the online image sets, bulk downloads are not easily done, images are downsampled to low resolution, and the filenames are not standardized, requiring significant manual effort to re-organize and collate them for a particular project. Adding further complications to data modularity, taxonomic data have often become partially obsolete.

Isolated fossil leaves present an additional set of challenging problems (e.g., Wilf 2008), including incomplete preservation, morphological convergence, and the well-known legacy of innumerable taxonomic misidentifications in older publications (see Dilcher 1971, 1974; Hill 1982). Numerous high-quality systematic treatments have become available for many leaf-fossil taxa, especially over the last few decades, but the images are dispersed across publications and are usually of low resolution. An increasing number of images of vouchered fossil-leaf collections is available online from natural history museums. Examples include aggregator sites such as GBIF (gbif.org) and individual institutions such as the Yale Peabody Museum, (https://peabody.yale.edu/collections/paleobotany), the Burke Museum (www.burkemuseum.org/collections-and-research/geology-and-paleontology/collections-database/images.php), the University of Colorado Boulder Museum of Natural History (https://www.colorado.edu/cumuseum/research-collections/paleontology/invertebrates-plants), and the UCMP (https://UCMPdb.berkeley.edu). Nevertheless, museum servers and project sites (e.g., Traiser et al. 2018) usually retain the taxonomy as published, which is vital for the nomenclatural stability of type specimens but well known to be problematic, especially for the many older collections that have not been revised under modern standards. All these issues make it very difficult for researchers, students, and non-specialists to form a reliable base of knowledge about fossil-leaf identification and have perhaps engendered an overreliance on methods that do not require taxonomy at all, such as leaf morphotyping (see Wilf 2008).

**Figure 1.**
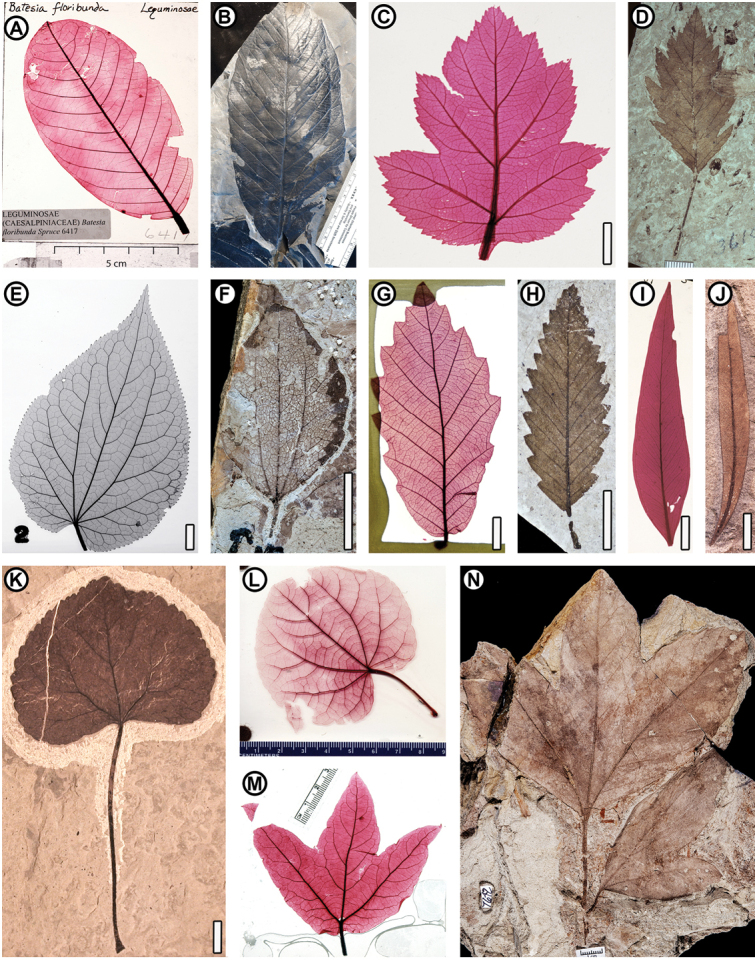
Selected image pairs of confamilial extant and fossil (see Appendix 1) leaves from the dataset **A***Batesiafloribunda* Spruce ex. Benth. (Fabaceae), NCLC-W 6417, showing typical layout of a cleared-leaf slide with original annotations (other examples are cropped in this figure); source voucher Froes 12074, DS 291771 (at CAS), Amazonas, Brazil **B**Fabaceae sp. CJ1, SGC-ICP-10173; Cerrejón mine, middle-late Paleocene of Guajira Peninsula, Colombia **C***Crataegusviridis* L. (Rosaceae), NCLC-W 11951b; H. Meyer s/n (collected 1974, no other voucher), cultivated, California, USA **D***Crataeguscopeana* (Rosaceae), UCMP 3610; Florissant, late Eocene of Colorado, USA; H. Meyer photograph number 0420 **E***Tetracentronsinense* Oliv. (Trochodendraceae), S. Wing negative 71-002; E.H. Wilson 659, US 599036, Szechuan, China **F***Ziziphoidesflabellum* (Trochodendraceae), USNM 560134; Mexican Hat, early Paleocene of Montana, USA **G***Quercusprinus* L. (Fagaceae), NCLC-W 6137; H. Foster 8223, US 1730249, Florida, USA **H***Fagopsislongifolia* (Fagaceae), FLFO 003432A; Florissant, late Eocene of Colorado, USA **I***Eucalyptusastringens* (Maiden) Maiden (Myrtaceae), NCLC-W 10489; J.H. Maiden (9 November 1909), Western Australia, UC 437518 **J***Eucalyptusfrenguelliana* (Myrtaceae), MPEF-Pb 2344; Laguna del Hunco, early Eocene of Chubut, Argentina **K***Cercidiphyllumobtritum* (Cercidiphyllaceae), DMNH 25061; Republic, early Eocene of Washington, USA **L***Cercidiphyllumjaponicum* Siebold & Zucc. ex J.J.Hoffm. & J.H.Schult.bis (Cercidiphyllaceae), Axelrod cleared leaf 166; UCMP (no other voucher) **M***Platanusracemosa* Nutt. (Platanaceae), NCLC-H 6631; Handel s/n (collected 1985, no other voucher), California, USA **N***Erlingdorfiamontana* (compound-leaved Platanaceae), DMNH 7642; Hell Creek Formation, Late Cretaceous of North Dakota, USA. Scale bars: centimeters as labeled (**A, B, L, M**); 1 cm when not labeled (**C–K, N**).

Here, we meet the community need for a specialized dataset of leaf images by consolidating a set of original-resolution photographs of vouchered extant and fossil specimens (Fig. 1, Table 1), primarily of angiosperms, vetted to family level and relabeled to user-friendly filenames, into an open-access archive in a single, standard file format (jpeg, at minimum possible compression). A principal goal, based on many years of practical experience using leaf-image datasets in our research, is maximum and sustained ease of use with rapid access to the entire library. Thus, instead of creating an interactive database that may become obsolete and limit resolution or user flexibility, we simply provide the image files in labeled folders that can easily be downloaded, then viewed and searched using any visual browser (e.g., Adobe Bridge, Adobe Lightroom, Windows Explorer) on any suitable device, such as a personal computer.

**Table 1.** Summary of component datasets.

Collection	Collection type	#Images	#Families	#Genera, approx.	#Species, approx.	Repository	Collection numbers†	Other data and images‡
NCLC-Wolfe	cleared leaves	16,249	267	3,893	12,439	USNM	secondary	http://clearedleavesdb.org
NCLC-Hickey	cleared leaves	6,861	313	1,678	5,723	USNM	secondary	https://collections.peabody.yale.edu/pb/nclc
Axelrod Cleared Leaves	cleared leaves	832	89	270	641	UCMP	primary	https://UCMPdb.berkeley.edu/photos/cleared_leaf.html
Wing X-Rays	x-ray negatives	2,234	26	416	890	USNM	secondary	n/a
**Total extant**		26,176	354	4,573	17,385			
Florissant, Meyer et al. (2008) project	fossil leaves	666	23	47	73	several	secondary	https://flfo-search.colorado.edu
Florissant, FLFO	fossil leaves	2,654	21	40	70	FLFO	primary	https://www.flickr.com/photos/155340198@N06
General fossil collection	fossil leaves	756	39	93	135	several	primary	n/a
**Total fossil**		4,076	48	129	222			

Abbreviations: FLFO, Florissant Fossil Beds National Monument. NCLC, National Cleared Leaf Collection. UCMP, University of California Museum of Paleontology. USNM, National Museum of Natural History, Smithsonian Institution. †As used in records specific to these collections and our image filenames. Secondary inventory numbers are those assigned by the creators of collections that were assembled from several primary collections. Examples are the cleared and x-rayed leaf samples physically gathered from primary herbarium sheets and the Meyer et al. (2008) Florissant photographic collection of specimens housed at numerous primary repositories. The Meyer et al. secondary (photograph) numbers have an informal “CU” prefix added here to the filenames, merely to distinguish them easily from the FLFO set in searches and not to indicate primary repository. See text for more details. ‡Images, specimen inventories, and other supporting data are available in the Figshare item accompanying this article: https://doi.org/10.25452/figshare.plus.14980698. Fossil taxa and references are listed in Appendix 1.

The full image dataset and supporting data files are available open-access for download in a single Figshare Plus data collection at https://doi.org/10.25452/figshare.plus.14980698 (hereafter, “the Figshare archive”). The components described below are summarized in Table 1, along with relevant online resources where many of the specimens can already be searched, usually at lower resolution. Although individual linkage of each specimen with online resources would be desirable, it is highly impractical at present because the necessary tags and lookup tables have never been compiled and vetted for most of the collections used here. For readability, we use “leaves” to refer to all specimens discussed here, whether they are leaves, leaflets, or other plant organs that are included in small numbers.

## ﻿Cleared and x-rayed leaves

The cleared and x-rayed leaf-image collections included here were chosen for availability of a large number of botanically diverse, high-quality images, accessible voucher data, and open-access re-use permissions. The collections primarily represent non-monocot (“dicot”) angiosperm leaves, with minor representation of monocots, other vascular plant groups, and non-foliar plant organs. Several other large cleared and x-rayed leaf collections exist (see Introduction) but were not used in the dataset presented here for various reasons. For example, the significant cleared-leaf atlas series by Klucking (1986–2003) was manually scanned, cropped, and made into a dataset as part of a machine-learning study (Wilf et al. 2016); however, that dataset is not retained here due to comparatively low resolution, moiré patterns, and other artifacts of printing (Wilf et al. 2016). In addition, the University of California Berkeley collection of over 800 vouchered cleared leaves (distinct from the Axelrod collection) has not been included here because it has not yet been digitized.

A master inventory of the 26,176 images of cleared and x-rayed specimens from >4,500 genera and >17,300 extant species in 354 plant families (Table 1) is provided in the accompanying Figshare archive. A small number of specimens are represented by multiple images, such as close-ups or lighting variants. Taxonomic fields include the family, genus, and species as provided in the respective collection catalog, with additional fields for updated Angiosperm Phylogeny Group (APG) family and order (APG IV 2016). Taxonomic and geographic coverage are uneven, constrained by the general availability of herbarium materials to the creators of the collections (similar issues occur even in recent, large herbarium-image datasets; e.g., de Lutio et al. 2021). Eight families are represented by more than 1,000 images each (Fabaceae, Sapindaceae, Rosaceae, Fagaceae, Annonaceae, Rubiaceae, Ulmaceae, and Malvaceae), whereas 173 families have fewer than ten images apiece. Photographs were taken by many people (see Acknowledgments) over an extended period of time and at different institutions, with a wide variety of cameras and methods that we do not attempt to detail; however, the original camera or scanner EXIF (Exchangeable Image File Format) metadata remain embedded in most of the images and are viewable in standard file browsers. We have also maintained the original pixel resolution and image dimensions in all photographs.

Catalog numbers of cleared or x-rayed leaves in the master inventory (available in the accompanying Figshare archive) refer to a unique glass slide (for the cleared leaves) or a film-negative number (for the x-rays) used to organize the respective collection, as designated by the creator of the collection. The catalog numbers of the cleared and x-rayed leaf collections are usually secondary, i.e., specific to the collection but linked in museum records (as legacy data and thus without hyperlinks) to a primary source voucher at a herbarium (Table 1). Thus, the collection-specific secondary numbers are usually the information needed to search the specimens online (using resources listed in Table 1 and further described below) or in paper catalogs to locate the primary source-voucher data. In some cases there is no herbarium or other voucher besides the mounted slide, and then the curated cleared-leaf specimen, usually specially collected for the purpose, is a primary collection. We also provide in the accompanying Figshare archive a catalog file containing the voucher data for the Wing X-Ray Collection, for which data are not otherwise available online. In publications, specimens should be formally cited by primary voucher as well as secondary catalog number if possible (see Fig. 1).

Family and order updates were done iteratively by first doing automatic lookups to family of the catalog genera and species, using the tables provided in The Plant List (www.theplantlist.org) and its successor, World Flora Online (WFO; www.worldfloraonline.org; Borsch et al. 2020). These resources include standardized lookups to family and order for most generic and species names, modified slightly to include a few taxa not listed in WFO. Failed lookups were flagged and corrected manually. Most lookup failures resulted from typographical errors of generic and species names in the catalog data, and these were manually corrected. Others resulted from genera not being listed or having ambiguous or unverified family status in WFO; these taxa were then manually vetted using other standard resources such as Tropicos (www.tropicos.org), the International Plant Names Index (www.ipni.org), and the Angiosperm Phylogeny Website (www.mobot.org/MOBOT/research/APweb). For consistency, the WFO was designated as the priority lookup for conflicting results among taxonomic databases.

For reference, we note other online resources for batch-vetting plant names that we did not use, including Taxonomic Names Resolution Service (tnrs.iplantcollaborative.org), taxize (github.com/ropensci/taxize), and the Kew Vascular Plant Families and Genera database (data.kew.org/vpfg1992/vascplnt.html). In addition, an automated tool, the WORLDFLORA R package, is now available for batch lookups from the WFO taxonomic backbone file (Kindt 2020), although this would not have resolved the large number of taxa with uncertain status in WFO that required manual vetting.

Due to the intensive labor that would be required to update the large number of names below family level, even with the aid of batch services, and the emphasis here on family-level vetting, generic and species names were for the most part not updated except to correct misspellings that would hinder future lookups. A full vetting below family level would also require manually consulting and hyperlinking all the primary herbarium records to check for new determinations, a process of several years. However, any user can easily find taxa of interest using the specimen list provided (accompanying Figshare archive) and access updated nomenclature and voucher data using the resources listed.

The resulting master inventory of cleared and x-rayed leaves was manually inspected repeatedly to eliminate variant spellings and other inconsistencies, until no more were found. Even after this stage, many issues remained from duplicate and corrupt files, invalid paths, labeling errors, ghost folders of problem images, and other common legacy database errors. Automated and reproducible data analysis and cleaning was done (by J. Rose and R. Saha) largely in Jupyter Notebooks and scripted in Python. In an iterative process, we used the Pandas library to load, sort, and filter the dataset in the form of a table, mapping metadata values in each column to unique specimens in each row. From there, we verified each file path’s full compliance with a pair of requirements, namely that it be both (a) a unique absolute path, and (b) a valid path specifying an existing, uncorrupted image file that can be successfully opened and closed. Rows that failed this test were flagged and taken out for manual review.

Further file path cleaning included the use of a fuzzy matching algorithm, through which all possible matches between a flagged query file path *q* and a possible near-duplicate reference path *f_R_* were compared by calculating the Levenshtein Distance (e.g., https://xlinux.nist.gov/dads/HTML/Levenshtein.html). This distance serves as a measure of the character-level similarity between two strings, from which all pairs are sorted in order of decreasing similarity to the flagged file *q*. Several duplicated source files that had evaded detection in previous stages were identified in this way, by manually scanning the top few most similar matches and searching for signs of typos. This procedure for automating the identification of the most likely near-duplicate strings allowed us to automatically verify that none of the tens of thousands of species in thousands of genera, hundreds of families, and dozens of orders included any artificial categories created by a misspelling. An example could be two samples from the same family, where one’s family was spelled “Fabaceae” (correct), whereas the other was accidentally entered as “Fabeceae.” This is an easy typo to miss, but it can skew downstream analyses.

Once all taxonomic and archival fields were validated, we assigned each sample a new filename that accomplishes both (a) directly encoding multiple levels of metadata into human-readable format within the filename, and (b) allowing easy sorting and searching of files on disk, without any additional alterations or struggling with a full relational database. The new filename format is constructed in the form: “Family_Genus_species_Collection_Catalog number”. This user-friendly format facilitates, for the first time, rapid alphabetic sorting, visual inspection, and searching of all the merged images from multiple sources in standard personal-computer windows and visual browsers. In the filenames, as just described, the family is updated to APG standard according to World Flora Online and other resources, whereas the genus and species fields are usually not updated except to correct spelling errors, especially those that could cause lookup failures.

### ﻿National cleared leaf collection – NCLC-W and NCLC-H

The National Cleared Leaf Collection is derived from parallel, broadly collaborative efforts supervised by the late Drs. Jack A. Wolfe (NCLC-W) and Leo J. Hickey (NCLC-H), beginning in the late 1960s. The NCLC is the world’s largest and most phylogenetically comprehensive assembly of cleared, stained, and mounted leaves sampled primarily from vouchered herbarium sheets. The collections underpinned the scientists’ research on fossil leaves and leaf architecture, including their landmark evolutionary survey (Hickey and Wolfe 1975). Hickey and Wolfe (1975) reported that the clearing techniques they used were those of Foster (1952), as adapted by Hickey (1973); Dilcher (1974) further described the techniques of Hickey and Wolfe. More recent work has improved the methods for clearing and mounting leaves without deterioration and provided historical methods reviews (Vasco et al. 2014; García-Gutiérrez et al. 2020). The Wolfe and Hickey cleared-leaf collections, kept separately during the scientists’ lifetimes and without any intention to merge them to our knowledge, are now curated together in the Division of Paleobotany, Department of Paleobiology, NMNH as the National Cleared Leaf Collection, constituting a monumental resource for leaf architecture that is combined here digitally for the first time. Physically, the two sub-collections are adjacent but not merged because Wolfe and Hickey used somewhat different family delimitations as they assembled their collections, and these are retained in the organization of the slides at NMNH (their systems were standardized and merged digitally for this contribution, as described earlier). The slides are organized alphabetically by family within each sub-collection.

The Wolfe contribution (NCLC-W) is the larger of the two parts, comprising over 18,000 specimens, from which 16,249 images are available here (Table 1). As described at the Cleared Leaf Image Database website (http://clearedleavesdb.org), the largest contributing source for NCLC-W was the University of California Herbarium (UC), Berkeley. Other significant sources were the California Academy of Sciences (CAS; including the Dudley Herbarium, DS, formerly of Stanford University), the Herbarium of the Arnold Arboretum (A) of the Harvard University Herbaria, the Missouri Botanical Garden (MO), the New York Botanical Garden (NY), the Field Museum of Natural History (F), and the National Herbarium of the Smithsonian Institution (US). Wolfe kept his collection for many years as a core reference for his voluminous body of work on fossil angiosperm leaves (see Upchurch et al. 2007), first at the United States Geological Survey (USGS) in Menlo Park, then at USGS Denver. Various photographic projects to document the collection advanced during the 1980s and 1990s, though none of these was published.

Following Dr. Wolfe’s retirement in 1992, S. Wing supervised the moving and curation of the cleared-leaf collection from Denver to NMNH, as well as, after Dr. Wolfe’s passing in 2005, a small portion of the collection that Wolfe had kept in his emeritus position at the University of Arizona. The collection was re-assembled, loaded into NMNH cabinetry, partially repaired, photographed, and placed under curation in the Division of Paleobotany, Department of Paleobiology, NMNH, officially as NCLC-W. A registry kept on paper by Dr. Wolfe and his team, containing the herbarium voucher data for all slides, is also kept with the collection; the registry was professionally transcribed into a digital format, then updated and corrected by E. González-Akre and several other Smithsonian staff members and volunteers. The photographs used in this contribution were made by another large group of Smithsonian staff and volunteers (see Acknowledgments). Most slides have approximately the same physical dimensions, although some are oversize to accommodate large leaves; scale bars are included on most photographs (e.g., Fig. 1A). The photographs and collections data for NCLC-W were separately archived several years ago in the Cleared Leaves Image Database (http://clearedleavesdb.org; Das et al. 2014), also under open-access but at lower resolution than we provide here. We refer the reader to that useful platform to look up primary specimen metadata online using Wolfe’s (secondary; Table 1) catalog numbers, including the herbarium-voucher data. Exact nomenclature may vary from what is presented here, following our separate vetting process.

The NCLC-W has been used extensively as a reference library, especially by paleobotanists; one notable example is its service as a principal reference for identifying leaf fossils from the oldest Neotropical paleorainforests, the Paleocene Cerrejón and Bogotá formation floras of Colombia (Herrera et al. 2008; Wing et al. 2009; Carvalho et al. 2021a, 2021b). Many images from NCLC-W (and NCLC-H) were used to illustrate leaf characters in the Manual of Leaf Architecture (Ellis et al. 2009), and the collection was used in a study of leaf rank and areole size (Green et al. 2014). A selection of more than 5,000 NCLC-W images was used for training and testing for family recognition as part of a machine-learning study that also included computer-marked heat maps, showing diagnostic regions for machine identification (Wilf et al. 2016).

Professor Leo J. Hickey supervised the assembly of a parallel cleared-leaf collection to Wolfe’s during his time as curator of paleobotany at NMNH (Wing et al. 2014), comprising more than 7,000 slides, from which 6,861 images are included here (Table 1). Dr. Hickey made a successful effort to sample complementary taxa to Dr. Wolfe, thus increasing the combined diversity of their collections considerably (Table 1). Hickey targeted a larger number of herbaceous taxa, partly reflecting his interest in herbaceous early angiosperms (e.g., Taylor and Hickey 1992). Nearly all specimens were sampled at US, with minor contributions from MO, NY, and several other herbaria, along with a small amount of freshly sampled or fluid-preserved material. Dr. Hickey borrowed the collection that he made when he relocated to the Yale Peabody Museum of Natural History (YPM) in 1982. Web access to images of NCLC-H and additional information about the collection are still provided by the Yale Peabody Museum (https://collections.peabody.yale.edu/pb/nclc/), where slides were imaged and inventoried by a large team (see Acknowledgments) under the direction of Drs. Hickey and S. Hu. The same photographs are aggregated here as summarized in the master inventory (available in the accompanying Figshare archive), and full metadata and source voucher information for each slide are available at https://collections.peabody.yale.edu/pb/nclc and from YPM staff. In NCLC-H, primary herbarium-voucher data are usually visible in the photographs on labels that were mounted with the leaves. The physical size of the slides varies, and scale bars are included on most photographs. Following Dr. Hickey’s passing in 2013, NCLC-H was returned to NMNH, where it is now curated in the Division of Paleobotany, Department of Paleobiology, adjacent to NCLC-W as just mentioned.

### ﻿Axelrod cleared leaf collection

The Daniel I. Axelrod Cleared Leaf Collection at UCMP includes about 1,300 specimens that are in exceptionally good condition, compared with the NCLC, because the late Dr. Axelrod (Barbour et al. 1998) mounted them in plexiglass with a medium, possibly clear epoxy, that has remained clear for over 50 years. The slides mostly represent the California flora. They are a self-standing primary collection not linked to herbarium vouchers, and only general locality data are given on the slide labels, but nevertheless the material comprises a well-curated museum collection with good preservation and high image quality in the photographs. The UCMP has provided the Axelrod images online for many years through several portals linked from the UCMP Cleared Leaf Collection web page (https://UCMP.berkeley.edu/collections/paleobotany-collection/UCMP-cleared-leaf-collection). Scale bars are included in all photographs, of which 832 are used here (Table 1). A selection of images from the Axelrod collection was used for training and testing of automatic leaf recognition in the Wilf et al. (2016) machine-learning study.

### ﻿Wing X-ray collection

In the early 1990s, S. Wing developed an x-ray scanning technique (Wing 1992) and used it to capture leaf and other organ images of selected families on large-format (8 by 10 inches, or 20.3 by 25.4 cm) x-ray negatives. The specimens are mostly from US, along with a variety of other herbaria and living collections; the negatives are now archived in the Division of Paleobotany, Department of Paleobiology, NMNH, and a separate data item is made available in the accompanying Figshare archive that matches the negative numbers (preserved in the current filenames) with their vouchers. The 1200-dpi cropped scans of the negatives by S. Wing are made available here digitally for the first time. Although the images lack embedded scales, the direct contact method of imaging with x-rays means that the images on physical negatives are the same size as the original specimens, and the negatives were scanned 1:1 as well. Thus, measurements can be made directly from the images or calibrated, if needed, using the post-crop image dimensions in the image metadata. Grayscale values of the scanned negatives were batch-inverted to positive here (easily reversed with a second inversion), to provide light backgrounds and improve comparability with the other image sets. The reverse grayscale tends to accentuate the visual impact of large differences in exposure caused by variation in leaf density; however, we found that standard image level and contrast adjustments are sufficient to make fine details more visible when needed. The collection includes a sizable number of x-rays of reproductive organs, especially Sapindaceae fruits, which are retained here for their general interest.

## ﻿Fossil leaves

We provide 4,076 vouchered leaf-fossil images of specimens that are assigned to family level, in total covering 44 angiosperm and four non-angiosperm families from a variety of sites in the AmeriCAS that are well known to the authors (Table 1; Appendix 1). Although far from comprehensive, this image set nevertheless covers at least a majority of angiosperm families that are reliably known in the fossil record from nearly-complete leaf remains; it provides a starter set both for comparative learning in angiosperm paleobotany and training machine-learning algorithms.

Unlike the images from the cleared and x-rayed collections, which were not adjusted except for cropping of the x-rays, the fossil-leaf images were all manually and reversibly rotated, close-cropped, and contrast- and temperature-adjusted (all whole-image adjustments, other than cropping) in Adobe Camera Raw so that they are approximately similar in relative frame alignment and overall contrast, with emphasis on making vein features visible (for some photographs taken on early-model digital cameras with barrel distortion in macro mode, the lens distortion was corrected manually using Adobe Camera Raw). This procedure minimizes strong distractors such as rock matrix for machine learning of fossil leaves, an interest of several of the authors (Wilf et al. 2016), and we found that it also enhances human learning for the same reason, by increasing visual comparability of the leaf features and eliminating distractors and variable orientation. In all CASes, we have maintained the full pixel resolution and (post-crop) dimensions of the original image and resaved processed images from Camera Raw to jpeg format only once (usually with tiff format as a lossless intermediate step), using the minimum compression ratio to maintain image quality. A cost of this approach was removal of most of the scale bars. However, nearly all scaling information can be accessed if needed from online (usually much lower resolution but suitable for scaling) versions of the images or sets of uncropped originals that we have included where necessary (see General Collection, below). In addition, all physical voucher specimens can be accessed at their respective repositories. As for the cleared and x-rayed leaves, original camera or scanner EXIF data remain embedded in the image metadata.

The fossil set of 4,076 images is comprised of two parts (Table 1, Appendix 1): first, a concentrated collection of 3,320 images from a single prolific site, the late Eocene Florissant fossil beds of Colorado; and second, a smaller general collection of 756 images from a variety of latest Cretaceous and Paleogene sites in North and South America (Appendix 1; accompanying Figshare archive). Appendix 1 annotates and lists authorities and taxonomic references for the ca. 222 species used in the fossil dataset, and individual catalog numbers are embedded in the filenames of the images. Appendix 1 also lists references for site-specific collections that pertain directly to the specimens used here, if the latter are different from the taxonomic references. Some specimens are represented by multiple images, such as close-ups or lighting variations (but not by duplicate images), and many images of counterparts are included. Although the major target for the collection was “dicot” leaves, images of a few species of monocots, ferns, and conifers that were readily available were included to help seed future expansions. Several generic names that may be botanically doubtful are left in as-published or as-cataloged form (Appendix 1), but all included material is considered reliably placed at family level. Informal morphotypes are included if they have reliable features at family level. Filenames, as for the cleared and x-rayed leaves, embed taxonomy to enable rapid auto-sorting and searching in standard PC windows, followed by collection data.

### ﻿Florissant collection

The late Eocene Florissant Fossil Beds Lagerstätte of Colorado is known worldwide for its long history of collection and investigation, its outstanding diversity of plant and animal fossils, and its seminal role in the conservation movement (MacGinitie 1953; Evanoff et al. 2001; Meyer 2003; Leopold et al. 2008; Veatch and Meyer 2008; Leopold and Meyer 2012). Florissant’s diverse fossil flora has a long history of study, resulting in an exceptional level of taxonomic understanding (e.g., Lesquereux 1873, 1883; MacGinitie 1953; Manchester and Crane 1983; 1987; Manchester 1989a, 2001a; Jia and Manchester 2014; Herendeen and Herrera 2019). The late Harry D. MacGinitie’s (1953) landmark monograph of the Florissant flora was outstanding among comparable works of the time for the high quality and botanical accuracy of his descriptions and identifications (Manchester 2001a).

Among its many distinctions, the Florissant biota was one of the first large fossil assemblages of any kind to be photographed, cataloged comprehensively, and made openly available in an internet database (Meyer et al. 2008). This massive effort by H. Meyer and associates, beginning in the 1990s, has two components as described below. The Florissant images were manually filtered and prepared by X. Zou from an initial set of 13,691 images of plant, animal, and geological specimens, of which 7,798 are of plants and 6,122 are of leaves, from which we further filtered and prepared the 3,320 images used here of leaf specimens that can be confidently placed in a plant family (Appendix 1). Vetting to plant family followed Manchester (2001a) and other publications as listed in Appendix 1.

The first of two components of the Florissant image set (Table 1) comes from the Meyer et al. (2008) project to capture high-resolution images of all type, published, and related Florissant collections, representing 5,663 specimens of ca. 1800 fossil plant and animal species as described in >300 scientific papers from a total Florissant collection of ca. 50,000 specimens. Much of this material had never been illustrated or was illustrated poorly by modern standards. The fossils are held in about 15 museums around the world as listed by Meyer et al. (2008); the largest three Florissant type and published collections are at the Smithsonian National Museum of Natural History, the Harvard University Museum of Comparative Zoology (almost entirely insects and spiders), and the University of Colorado Museum of Natural History. The original photographs on Kodachrome slides are archived at Florissant Fossil Beds National Monument (FLFO), and they were scanned twice about 12–15 years apart to take advantage of improving technology, the second time at high resolution. The resulting image database of the more recent scans (Meyer et al. 2008) was hosted on a National Park Service server initially and then moved several years ago to the University of Colorado Museum of Natural History, where it can be searched online using the Florissant Fossil Beds Collection Search at https://flfo-search.colorado.edu. That website provides full specimen metadata and reduced-resolution image files (with scale bars), which can be searched using the secondary inventory (photograph) numbers from the Meyer et al. (2008) project that are here embedded in the filenames (Table 1). To help distinguish images in this collection from the others rapidly in searches, we have also attached an informal “CU” prefix (for the University of Colorado) to the secondary catalog numbers in the filenames.

The second component of the Florissant image collection provided here (Table 1) is a selection of fully digital images from the collections at Florissant Fossil Beds National Monument (primary acronym FLFO, specimen number embedded in the filename), assembled by H. Meyer and numerous interns and assistants. The images can also be searched and viewed (with scale bars but at lower resolution) by FLFO number on the park’s flickr page, located at https://www.flickr.com/photos/155340198@N06. The corresponding, full-resolution, uncropped images that were processed here from both Florissant image sets are archived at the University of Colorado Museum of Natural History and FLFO, respectively, and available on request to collections management.

### ﻿General collection

The general collection of 756 fossil leaf images provided here (Appendix 1; specimen data in the accompanying Figshare archive) draws from a set of Late Cretaceous to Eocene fossil floras from the AmeriCAS. The general collection diversifies the phylogenetic, preservational, temporal, and geographic coverage of the overall fossil-image dataset and forms a base to encourage other teams to make similar efforts. Repositories of the material are indicated in the filenames and supplemental data files in the accompanying Figshare archive; they include the Denver Museum of Nature & Science (repository acronym DMNH); Museo Paleontológico Egidio Feruglio (MPEF-Pb, Trelew, Argentina); National Museum of Natural History, Smithsonian Institution (USNM-PAL, abbreviated here as USNM, Washington, D.C.); Colombian Geological Survey and Colombian Petroleum Institute (combined as SGC-ICP, Bogotá, Colombia); Florida Museum of Natural History (UF, Gainesville); and University of California Museum of Paleontology (UCMP, Berkeley). Filenames embed the taxonomy as well as primary repository numbers or unique field numbers, if a formal repository number is not assigned (some MPEF, USNM specimens). In a few CASes, where more than one image of the same fossil is included (i.e., close-ups or unlabeled parts and counterparts), an informal tag is included in the filename in brackets to ensure uniqueness of filenames. Some material lacks formal taxonomy but is considered reliably identified at family level; there are also a few CASes of historic generic identifications considered incorrect and indicated by quotations (e.g., “*Acer*,” “*Ficus*”) but still assignable to a (often different) family (Appendix 1). Because very few fossils from the general collection are otherwise viewable online to check scale bars, we provide a parallel folder of the corresponding uncropped images in the accompanying Figshare archive.

Major contributions to the general collection are briefly listed here for paleobotanists, with additional taxonomic and occurrence references listed in Appendix 1. The fossils come from (1) a suite of latest Cretaceous (late Maastrichtian, Hell Creek Formation) and early Paleocene (early Danian, Fort Union Formation) sites from western North Dakota and South Dakota USA that have been used extensively for studies of the end-Cretaceous extinction (e.g., Johnson et al. 1989; Johnson 2002; Wilf and Johnson 2004); (2) the early Paleocene Salamanca Formation (early Danian) and Las Flores (late Danian) floras of Chubut, Argentina, known for diverse and well-preserved fossil plants and insect-feeding damage following the end-Cretaceous extinction (e.g., Iglesias et al. 2007, 2021; Clyde et al. 2014; Donovan et al. 2017; Stiles et al. 2020); (3) the early Paleocene (Danian, Fort Union Formation) Mexican Hat site in southeastern Montana, USA, known for diverse insect herbivory traces preserved in its fossil leaves (Wilf et al. 2006; Winkler et al. 2010; Donovan et al. 2014); (4) the middle-late Paleocene (Selandian-Thanetian, Cerrejón Formation) Cerrejón flora from the Guajira Peninsula, Colombia and Bogotá Formation flora of Sabana de Bogotá, central Colombia, together preserving the remains of the oldest known Neotropical rainforests (e.g., Doria et al. 2008; Herrera et al. 2008, 2019; Gómez-Navarro et al. 2009; Wing et al. 2009; Carvalho et al. 2011, 2021a, 2021b); (5) a suite of sites spanning the late Paleocene (Fort Union Formation) through early Eocene (Wasatch Formation and Little Mountain locality of the Green River Formation) of southwestern and northwestern Wyoming that have been used in many studies of floristic and plant-insect associational responses to climate change (e.g., Gemmill and Johnson 1997; Wilf et al. 1998, 2006; Wilf and Labandeira 1999; Wilf 2000; Donovan et al. 2014); (6) the early Eocene Laguna del Hunco Lagerstätte in Chubut, Argentina (Huitrera Formation), known for its outstanding diversity of fossil plants and animals, varied biogeographic connections, and large number of unique taxon occurrences for South America (e.g., Wilf et al. 2003, 2013, 2019; Gandolfo et al. 2011); (7) the late early Eocene flora of Republic, Washington (Wolfe and Wehr 1987; DeVore et al. 2005; Greenwood et al. 2016; Klondike Mountain Formation) and the middle Eocene Green River Formation flora (MacGinitie 1969; Smith et al. 2008) of Bonanza, Utah, specifically using images of field-censused collections at DMNH from both sites led by K. Johnson that were used previously for analyses of insect herbivory, fossil-leaf economics, and digital leaf physiognomy (Wilf et al. 2001, 2005b; Cariglino 2007; Royer et al. 2007; Peppe et al. 2011).

## ﻿Concluding remarks

The dataset presented here consolidates thousands of hours of labor by many people (see Acknowledgments) into a single accessible platform. Due to the extraordinary effort involved, it is unlikely that many new, large-scale cleared and x-rayed leaf collections will ever be assembled and digitally processed. Thus, the future prospects for significantly increasing the overall sample size and improving the coverage of taxonomy and geography in digital leaf-reference collections most likely lie elsewhere. The greatest potential appears to come from the advancing techniques for segmenting and enhancing leaf images from the enormous, widely available resource of digitized herbarium sheets (Hussein et al. 2020; Weaver et al. 2020), which have the significant additional advantage of direct linkage to the global data infrastructure for biodiversity (e.g., Bakker et al. 2020). To reach comparability with cleared leaves, segmented leaf images will require high pixel resolution, optimized contrast for the capture of venation details, and the careful retention of significant edge features such as the leaf margin. For leaf fossils, increasing the sample size of well-identified specimens is straightforward in principle but will require efforts far beyond the resources of a single collaboration. Thus, we plan a community initiative for this purpose.

We look forward to seeing the assembled image dataset catalyze advances in research, education, and outreach. The images and supporting data are available open-access on Figshare Plus at https://doi.org/10.25452/figshare.plus.14980698. Some mistakes are inevitable in a first-version database of this nature; please report any errors observed to the corresponding author. Corrections and updates may be applied to the Figshare archive under new version numbers; the version precisely corresponding to this article will remain preserved as version 1.0.

## ﻿Funding

Funding for this work came from NSF grants EAR-1925755, EAR-1925481, and EAR-1925552 (PW, TS, MAG, and others); DEB-1556666 and DEB-1556136 (PW, MAG, and others); and the National Park Service (HWM).

## ﻿Appendix 1

**Table:** Species list for fossil-leaf images.

Species	#Images	Source, age, region†	References
**Dryopteridaceae**			
*Dryopterisguyottii* (Lesquereux) MacGinitie	64	Florissant, late Eocene, Colorado, USA	MacGinitie 1953
**Cupressaceae**			
*Chamaecyparislinguaefolia* (Lesquereux) MacGinitie, *Chamaecyparis* sp.	100	Florissant, late Eocene, Colorado, USA	MacGinitie 1953; Manchester 2001a
*Sequoiaaffinis* Lesquereux, *Sequoia* sp.	153	Florissant, late Eocene, Colorado, USA	MacGinitie 1953; Manchester 2001a
**Pinaceae**			
*Pinusflorissantii* Lesquereux	9	Florissant, late Eocene, Colorado, USA	MacGinitie 1953; Manchester 2001a
*Pinusmacginitieii* Axelrod	1	Florissant, late Eocene, Colorado, USA	Axelrod 1986; Manchester 2001a
*Pinuswheeleri* Cockerell, *Pinus* sp.	55	Florissant, late Eocene, Colorado, USA	Cockerell 1908; MacGinitie 1953; Manchester 2001a
**Taxaceae**			
*Torreyageometrorum* (Cockerell) MacGinitie, *Torreya* sp.	9	Florissant, late Eocene, Colorado, USA	MacGinitie 1953; Manchester 2001a
**Araceae**			
Araceae sp. CJ80	2	Cerrejón mine, middle-late Paleocene, Guajira Peninsula, Colombia	Wing et al. 2009
*Limnobiophyllumscutatum* (Dawson) Krassilov	2	Florissant, late Eocene, Colorado, USA	see Manchester 2001a
*Montrichardiaaquatica* Herrera et al.	4	Cerrejón mine, middle-late Paleocene, Guajira Peninsula, Colombia	Herrera et al. 2008
*Petrocardiumcerrejonense* Herrera et al.	1	Cerrejón mine, middle-late Paleocene, Guajira Peninsula, Colombia	Herrera et al. 2008
*Petrocardiumwayuuorum* Herrera et al.	1	Cerrejón mine, middle-late Paleocene, Guajira Peninsula, Colombia	Herrera et al. 2008
**Arecaceae**			
Arecaceae spp. CJ67, CJ68	4	Cerrejón mine, middle-late Paleocene, Guajira Peninsula, Colombia	Gómez-Navarro et al. 2009; Wing et al. 2009
**Rhipogonaceae**			
*Ripogonumamericanum* R.J. Carpenter et al.	2	Laguna del Hunco, early Eocene, Chubut, Argentina	Carpenter et al. 2014
**Zingiberaceae**			
Zingiberaceae spp. CJ49, CJ65	3	Cerrejón mine, middle-late Paleocene, Guajira Peninsula, Colombia	Wing et al. 2009
**Adoxaceae**			
*Sambucusnewtoni* Cockerell	33	Florissant, late Eocene, Colorado, USA	MacGinitie 1953; Manchester 2001a
**Akaniaceae**			
*Akaniapatagonica* Gandolfo et al.	9	Laguna del Hunco, early Eocene, Chubut, Argentina	Gandolfo et al. 1988; Wilf et al. 2005a‡
**Altingiaceae**			
“*Acer*” *lesquereuxi* Knowlton	2	Little Mountain & Bonanza, early (LM) and middle (B) Eocene, Wyoming (LM) & Utah (B), USA	MacGinitie 1969; Johnson and Plumb 1995; Wilf et al. 2005b‡
**Anacardiaceae**			
Anacardiaceae sp. CJ34	4	Cerrejón mine, middle-late Paleocene, Guajira Peninsula, Colombia	Wing et al. 2009
Anacardiaceae sp. TY203	1	Laguna del Hunco, early Eocene, Chubut, Argentina	P. Wilf, unpubl. obs.
*Rhuslesquereuxi* Knowlton & Cockerell	21	Florissant, late Eocene, Colorado, USA	MacGinitie 1953; Manchester 2001a
*Rhusmalloryi* Wolfe & Wehr	5	Republic, early Eocene, Washington, USA	Wolfe and Wehr 1987; Wilf et al. 2005b‡; Flynn et al. 2019
*Rhusnigricans* (Lesquereux) Knowlton	27	Little Mountain & Bonanza, early and middle Eocene, Wyoming & Utah, USA	MacGinitie 1969; Wilf 2000‡; Wilf et al. 2001‡
*Rhusobscura* (Lesquereux) MacGinitie	22	Florissant, late Eocene, Colorado, USA	MacGinitie 1953; Manchester 2001a
*Rhusstellariaefolia* (Lesquereux) MacGinitie, *Rhus* sp.	175	Florissant, late Eocene, Colorado, USA	MacGinitie 1953; Manchester 2001a
Schmalzia (Rhus) vexans (Lesquereux) Cockerell	5	Florissant, late Eocene, Colorado, USA	MacGinitie 1953
**Apocynaceae**			
Apocynaceae sp. RR17	1	Wasatch Fm., early Eocene, Wyoming, USA	Wing 1998; Wilf 2000‡
**Atherospermataceae**			
*Atherospermophyllumguinazui* (E.W. Berry) C.L. Knight	16	Laguna del Hunco, early Eocene, Chubut, Argentina	Knight and Wilf 2013
**Berberidaceae**			
*Mahoniamarginata* (Lesquereux) Arnold	15	Florissant, late Eocene, Colorado, USA	MacGinitie 1953; Manchester 2001a
*Mahoniasubdenticulata* (Lesquereux) MacGinitie, *Mahonia* sp.	11	Florissant, late Eocene, Colorado, USA	MacGinitie 1953; Manchester 2001a
**Betulaceae**			
*Alnusparvifolia* (E.W. Berry) Wolfe and Wehr	33	Republic, early Eocene, Washington, USA	Wolfe and Wehr 1987; Wilf et al. 2005b‡
*Alnus* sp. RR14	1	Wasatch Fm., early Eocene, Wyoming, USA	Wilf 2000
*Betulaleopoldae* Wolfe and Wehr	4	Republic, early Eocene, Washington, USA	Wolfe and Wehr 1987; Wilf et al. 2005b‡
*Betula* sp. RP21	1	Republic, early Eocene, Washington, USA	Wilf et al. 2005b
*Corylites* spp. BB05, FW01	3	Fort Union Fm., several sites, late Paleocene, Wyoming, USA	Manchester and Chen 1996; Wilf 2000‡
*Paracarpinusfraterna* (Lesquereux) Manchester & Crane, *Paracarpinus* sp.	58	Florissant, late Eocene, Colorado, USA	Manchester and Crane 1987; Manchester 2001a
**Cannabaceae**			
*Celtisaspera* (Newberry) Manchester et al.§	3	Fort Union Fm., several sites, late Paleocene, Wyoming, USA	Wilf 2000‡; Manchester et al. 2002, 2018; Wilf et al. 2006‡
**Cercidiphyllaceae**			
*Archeampeloslobatocrenata* (Lesquereux) Doweld	2	Fort Union Fm., several sites, late Paleocene, Wyoming, USA	McIver and Basinger 1993; Wilf 2000‡; Wilf et al. 2006‡; Manchester 2014; Doweld 2016
*Cercidiphyllumobtritum* (Dawson) Wolfe and Wehr	12	Republic, early Eocene, Washington, USA	Wolfe and Wehr 1987; Wilf et al. 2005b‡
*Trochodendroidesgenetrix* (Newberry) Manchester	13	Fort Union Fm., several sites, early & late Paleocene, Montana, North Dakota, & Wyoming, USA	Wilf 2000‡; Johnson 2002‡; Wilf et al. 2006‡; Manchester 2014
**Cornaceae**			
*Beringiaphyllumcupanioides* (Newberry) Manchester et al.	3	SW Wyoming, several sites, late Paleocene, Wyoming, USA	Manchester et al. 1999; Wilf 2000‡; Wilf et al. 2006‡
*Brownieaserrata* (Newberry) Manchester & Hickey	6	Fort Union Fm., several sites, late Paleocene, Wyoming, USA	Wilf 2000‡; Wilf et al. 2006‡; Manchester and Hickey 2007
*Cornus* sp. RP62	3	Republic, early Eocene, Washington, USA	Wolfe and Wehr 1987; Wilf et al. 2005b‡
*Cornusswingii* Manchester et al.	2	Fort Union Fm., several sites, late Paleocene, Wyoming, USA	Wilf 2000‡; Wilf et al. 2006‡; Manchester et al. 2009
*Davidiaantiqua* (Newberry) Manchester	4	Fort Union Fm., several sites, late Paleocene, Wyoming, USA	Wilf 2000‡; Manchester 2002; Wilf et al. 2006‡
**Cunoniaceae**			
Cunoniaceae sp. SA020	13	Salamanca Fm., early Paleocene, Chubut, Argentina	Iglesias et al. 2007, 2021
Cunoniaceae sp. TY116	9	Laguna del Hunco, early Eocene, Chubut, Argentina	Wilf et al. 2005a; Merkhofer et al. 2015
**Ericaceae**			
Ericaceae sp. RP41	1	Republic, early Eocene, Washington, USA	Wilf et al. 2005b‡
Rhododendron sp. RP53	1	Republic, early Eocene, Washington, USA	Wilf et al. 2005b‡
**Euphorbiaceae**			
Euphorbiaceae sp. CJ24	4	Cerrejón mine, middle-late Paleocene, Guajira Peninsula, Colombia	Wing et al. 2009
*StillingiaCASca* Hickey	1	Wasatch Fm., early Eocene, Wyoming, USA	Hickey 1977; Wilf 2000‡
**Fabaceae**			
*Caesalpiniapecorae* Brown	9	Little Mountain & Bonanza, early and middle Eocene, Wyoming & Utah, USA	MacGinitie 1969; Wilf 2000‡; Wilf et al. 2001‡
*Caesalpinitesacuminatus* (Lesquereux) MacGinitie	13	Florissant, late Eocene, Colorado, USA	MacGinitie 1953; Manchester 2001a
*Caesalpinitescoloradicus* MacGinitie	21	Florissant, late Eocene, Colorado, USA	MacGinitie 1953; Manchester 2001a
*Cercisparvifolia* Lesquereux	23	Florissant, late Eocene, Colorado, USA	MacGinitie 1953; Jia and Manchester 2014
*Conzattiacoriacea* MacGinitie, Fabaceae sp.	21	Florissant, late Eocene, Colorado, USA	MacGinitie 1953
Fabaceae sp. SA045	1	Salamanca Fm., early Paleocene, Chubut, Argentina	Iglesias et al. 2007, 2021; Brea et al. 2008
Fabaceae sp. TY117	14	Laguna del Hunco, early Eocene, Chubut, Argentina	Wilf et al. 2005a
Fabaceae spp. CJ1, CJ19, CJ55	15	Cerrejón mine, middle-late Paleocene, Guajira Peninsula, Colombia	Wing et al. 2009; Herrera et al. 2019
Fabaceae spp. GR554, GR567	3	Little Mountain, early Eocene, Wyoming, USA	Wilf 2000
Fabaceae spp. morphotypes 2, 3	3	Cogua & Nemocón mines, late Paleocene, Cogua, Cundinamarca, Colombia	Herrera et al. 2019
*Gymnocladushesperia* (Brown) MacGinitie	1	Little Mountain, early Eocene, Wyoming, USA	MacGinitie 1969; Wilf 2000‡
*Leguminositeslespedezoides* MacGinitie	1	Florissant, late Eocene, Colorado, USA	MacGinitie 1953; Manchester 2001a
*Leguminositeslesquereuxiana* (Knowlton) Brown	6	Bonanza, middle Eocene, Utah, USA	MacGinitie 1969; Wilf et al. 2001‡
*Parvileguminophyllumcoloradensis* (Knowlton) Call & Dilcher	30	Little Mountain & Bonanza, early and middle Eocene, Wyoming & Utah, USA	Call and Dilcher 1994; Wilf 2000‡; Wilf et al. 2001‡
“*Prosopis*” *linearifolia* (Lesquereux) MacGinitie	23	Florissant, late Eocene, Colorado, USA	MacGinitie 1953; Manchester 2001a
*Robinialesquereuxi* (Ettingshausen) MacGinitie	39	Florissant, late Eocene, Colorado, USA	MacGinitie 1953; Manchester 2001a
**Fagaceae**			
*CAStaneadolichophylla* Cockerell	15	Florissant, late Eocene, Colorado, USA	MacGinitie 1953; Manchester 2001a
*CAStaneophyllumpatagonicum* Wilf et al.	17	Laguna del Hunco, early Eocene, Chubut, Argentina	Wilf et al. 2019
cf. *Quercus* sp. GR522	2	Little Mountain, early Eocene, Wyoming, USA	Wilf 2000
Fagaceae spp. RP060, RP154	2	Republic, early Eocene, Washington, USA	Manchester and Dillhoff 2004; Wilf et al. 2005b
*Fagopsislongifolia* (Lesquereux) Hollick, *Fagopsis* sp., cf. *Fagopsis* sp.	521	Florissant, late Eocene, Colorado, USA	Manchester and Crane 1983; Manchester 2001a
*Quercusdumosoides* MacGinitie	9	Florissant, late Eocene, Colorado, USA	MacGinitie 1953; Manchester 2001a
*Quercuslyratiformis* Cockerell	1	Florissant, late Eocene, Colorado, USA	MacGinitie 1953; Manchester 2001a
*Quercusmohavensis* Axelrod	9	Florissant, late Eocene, Colorado, USA	MacGinitie 1953; Manchester 2001a
*Quercusorbata* MacGinitie	11	Florissant, late Eocene, Colorado, USA	MacGinitie 1953; Manchester 2001a
*Quercusperitula* Cockerell	12	Florissant, late Eocene, Colorado, USA	MacGinitie 1953; Manchester 2001a
*Quercuspredayana* MacGinitie	8	Florissant, late Eocene, Colorado, USA	MacGinitie 1953; Manchester 2001a
*Quercusscottii* (Lesquereux) MacGinitie	37	Florissant, late Eocene, Colorado, USA	MacGinitie 1953; Manchester 2001a
*Quercusscudderi* Knowlton, *Quercus* sp.	46	Florissant, late Eocene, Colorado, USA	MacGinitie 1953; Manchester 2001a
**Grossulariaceae**			
*Ribeserrans* MacGinitie	3	Florissant, late Eocene, Colorado, USA	MacGinitie 1953; Manchester 2001a
**Hamamelidaceae**			
*Langeriamagnifica* Wolfe and Wehr	2	Republic, early Eocene, Washington, USA	Wolfe and Wehr 1987; Wilf et al. 2005b‡
**Hydrangeaceae**			
*Philadelphusminutus* MacGinitie	1	Florissant, late Eocene, Colorado, USA	MacGinitie 1953; Manchester 2001a
**Iteaceae**			
*Itea* sp. RP19	3	Republic, early Eocene, Washington, USA	Wolfe and Wehr 1987; Wilf et al. 2005b‡; Hermsen 2013
**Juglandaceae**			
*Caryalibbeyi* (Lesquereux) MacGinitie, *Carya* sp.	53	Florissant, late Eocene, Colorado, USA	MacGinitie 1953; Manchester 1987; 2001a
Juglandaceae sp. BB09	1	Bison Basin, late Paleocene, Wyoming, USA	Gemmill and Johnson 1997; Wilf 2000
Juglandaceae sp. GR519	1	Little Mountain, early Eocene, Wyoming, USA	Wilf 2000
*Juglandiphyllitesglabra* (R. Brown) Manchester and Dilcher	7	Fort Union Fm., several sites, early & late Paleocene, Montana & Wyoming, USA	Manchester and Dilcher 1997; Wilf 2000‡; Wilf et al. 2006‡
*Platycaryaamericana* Hickey	1	Wasatch Fm., early Eocene, Wyoming, USA	Wing and Hickey 1984; Manchester 1987; Wilf 2000‡
*PlatycaryaCAStaneopsis* (Lesquereux) Wing & Hickey	1	Wasatch Fm., early Eocene, Wyoming, USA	Wing and Hickey 1984; Manchester 1987; Wilf 2000‡
**Lauraceae**			
“*Ficus*” *planicostata* Lesquereux	1	Battleship, Maastrichtian, North Dakota, USA	Johnson 2002‡
Lauraceae sp. RR19	1	Wasatch Fm. several sites, early Eocene, Wyoming, USA	Wilf 2000
Lauraceae sp. SA010	17	Salamanca Fm., early Paleocene, Chubut, Argentina	Iglesias et al. 2007, 2021
Lauraceae sp. TY084	21	Laguna del Hunco, early Eocene, Chubut, Argentina	Wilf et al. 2005a
Lauraceae spp. CJ5. CJ22	8	Cerrejón mine, middle-late Paleocene, Guajira Peninsula, Colombia	Wing et al. 2009
Lauraceae spp. FW02, FW03, FW28	6	Fort Union Fm., several sites, late Paleocene, Wyoming, USA	Wilf 2000
*Laurophyllumpiatnitzkyi* E.W. Berry	12	Salamanca & Peñas Coloradas fms., early Paleocene, Chubut, Argentina	Iglesias et al. 2007, 2021‡
*Linderacoloradica* MacGinitie	4	Florissant, late Eocene, Colorado, USA	MacGinitie 1953; Manchester 2001a
*Linderavarifolia* MacGinitie	18	Little Mountain, early Eocene, Wyoming, USA	MacGinitie 1969; Wilf et al. 2001‡
*Marmarthiapearsoni* K. Johnson	5	Hell Creek Fm., several sites, Maastrichtian, North Dakota, USA	Johnson 1996, 2002
*Marmarthiatrivialis* K. Johnson	2	Madeline’s Bank, Hell Creek Fm., Maastrichtian, North Dakota, USA	Johnson 1996, 2002
*Persitesargutus* Hickey	11	Fort Union Fm., several sites, late Paleocene, Wyoming, USA	Hickey 1977; Wilf 2000‡; Wilf et al. 2006‡
“*Sassafras*” *hesperia* E.W. Berry	2	Florissant, late Eocene, Colorado, USA	MacGinitie 1953; Manchester 2001a
“*Sassafras*” *hesperia* E.W. Berry	6	Republic, early Eocene, Washington, USA	Wolfe and Wehr 1987; Wilf et al. 2005b‡
**Magnoliaceae**			
*Liriodendritesbradacii* K. Johnson	1	Madeline’s Bank, Hell Creek Fm., Maastrichtian, North Dakota, USA	Johnson 1996, 2002
**Malvaceae**			
“*Dombeya*” *novi-mundi* Hickey	1	Wasatch Fm., several sites, early Eocene, Wyoming, USA	Hickey 1977; Wilf 2000‡; Carvalho et al. 2011
Malvaceae sp. TY023	16	Laguna del Hunco, early Eocene, Chubut, Argentina	Wilf et al. 2005a; Wilf 2008
Malvaceae spp. CJ25, CJ36	3	Cerrejón mine, middle-late Paleocene, Guajira Peninsula, Colombia	Wing et al. 2009; Carvalho et al. 2011
*Malvaciphyllummacondicus* M. Carvalho	6	Cerrejón mine, middle-late Paleocene, Guajira Peninsula, Colombia	Wing et al. 2009; Carvalho et al. 2011
*Tiliajohnsoni* Wolfe & Wehr	1	Republic, early Eocene, Washington, USA	Wolfe and Wehr 1987; Wilf et al. 2005b‡
*Triumfettaovata* MacGinitie	4	Little Mountain, early Eocene, Wyoming, USA	MacGinitie 1969; Wilf 2000‡
**Meliaceae**			
*Cedrelalancifolia* (Lesquereux) Brown	35	Florissant, late Eocene, Colorado, USA	MacGinitie 1953; Manchester 2001a
Meliaceae sp. CJ2	3	Cerrejón mine, middle-late Paleocene, Guajira Peninsula, Colombia	Wing et al. 2009
**Menispermaceae**			
Menispermaceae sp. GR507	1	Little Mountain, early Eocene, Wyoming, USA	Wilf 2000
*Menispermitescerrejonensis* Doria et al.	6	Cerrejón mine, middle-late Paleocene, Guajira Peninsula, Colombia	Doria et al. 2008
*Menispermitescordatus* Doria et al.	1	Cerrejón mine, middle-late Paleocene, Guajira Peninsula, Colombia	Doria et al. 2008
*Menispermiteshorizontalis* Doria et al.	1	Cerrejón mine, middle-late Paleocene, Guajira Peninsula, Colombia	Doria et al. 2008
*Wilkinsoniphyllummenispermoides* Jud et al.	1	Salamanca Fm., early Paleocene, Chubut, Argentina	Iglesias et al. 2007, 2021; Jud et al. 2018
**Monimiaceae**			
*Monimiophyllumcallidentatum* C.L. Knight	1	Laguna del Hunco, early Eocene, Chubut, Argentina	Knight and Wilf 2013
**Myricaceae**			
*Comptoniacolumbiana* Dawson	1	Republic, early Eocene, Washington, USA	Wolfe and Wehr 1987; Wilf et al. 2005b‡
**Myrtaceae**			
*Eucalyptusfrenguelliana* Gandolfo & Zamaloa	13	Laguna del Hunco, early Eocene, Chubut, Argentina	Gandolfo et al. 2011; Hermsen et al. 2012
*Eugeniaarenaceaeformis* (Cockerell) MacGinitie	16	Florissant, late Eocene, Colorado, USA	MacGinitie 1953; Manchester 2001a
Myrtaceae sp. TY41	1	Laguna del Hunco, early Eocene, Chubut, Argentina	Wilf et al. 2005a
*Syzygioidesamericana* (Lesquereux) Manchester et al.	5	Little Mountain, Bonanza, & Wasatch Fm., early and middle Eocene, Wyoming & Utah, USA	Manchester et al. 1998; Wilf 2000‡; Wilf et al. 2005b‡
**Platanaceae**			
*Erlingdorfiamontana* (Brown) K. Johnson	15	Hell Creek Fm., several sites, Maastrichtian, North Dakota, USA	Johnson 1996, 2002
*Grewiopsissaportana* Lesquereux	8	Hell Creek Fm., several sites, Maastrichtian, North Dakota, USA	Johnson 2002‡
*Leepiercieapreartocarpoides* K. Johnson	5	Hell Creek Fm., several sites, Maastrichtian, North Dakota, USA	Johnson 1996, 2002
*Macginitieagracilis* (Lesquereux) Wolfe and Wehr	4	Fort Union Fm., several sites & Republic, late Paleocene, early Eocene, Washington & Wyoming, USA	Wolfe and Wehr 1987; Wilf 2000‡
*Macginitieawyomingensis* (Knowlton & Cockerell) Manchester	9	Little Mountain & Bonanza, early and middle Eocene, Wyoming & Utah, USA	Manchester 1986; Wilf 2000‡; Wilf et al. 2005b‡
*Platanitesmarginata* (Lesquereux) K. Johnson	7	Hell Creek Fm., several sites, Maastrichtian, North Dakota, USA	Johnson 1996, 2002
*Platanitesraynoldsii* (Newberry) Manchester	21	Fort Union Fm., several sites, early & late Paleocene, Montana, North Dakota, & Wyoming, USA	Wilf 2000‡; Johnson 2002‡; Wilf et al. 2006‡; Manchester 2014
*Platanus* sp. GR506	1	Little Mountain, early Eocene, Wyoming, USA	Wilf 2000
**Proteaceae**			
*Lomatiaoccidentalis* (E.W. Berry) Frenguelli	32	Laguna del Hunco, early Eocene, Chubut, Argentina	González et al. 2007
*Lomatiapreferruginea* E.W. Berry	9	Laguna del Hunco, early Eocene, Chubut, Argentina	González et al. 2007
Proteaceae gen. et sp. indet. (sp. TY208)	1	Laguna del Hunco, early Eocene, Chubut, Argentina	González et al. 2007
**Rhamnaceae**			
*Hovenia* cf. *H.oregonensis* Meyer & Manchester	1	Wasatch Fm., several sites, early Eocene, Wyoming, USA	Meyer and Manchester 1997; Wilf 2000‡
Rhamnaceae sp. TY025	15	Laguna del Hunco, early Eocene, Chubut, Argentina	Wilf et al. 2005a
*Rhamnicacleburnii* (Lesquereux) Doweld	1	Battleship, Maastrichtian, North Dakota, USA	McIver and Basinger 1993; Johnson 2002‡; Doweld 2016
*Suesseniagrandensis* Jud et al.	4	Rancho Grande, early Paleocene, Chubut, Argentina	Jud et al. 2017 (source of images used)
*Zizyphusflorissantii* (Lesquereux) MacGinitie.	11	Florissant, late Eocene, Colorado, USA	MacGinitie 1953; Manchester 2001a
**Rosaceae**			
*Amelanchierscudderi* Cockerell, *Amelanchier* sp.	10	Florissant, late Eocene, Colorado, USA	MacGinitie 1953; Manchester 2001a
*Cercocarpusmyricaefolius* (Lesquereux) MacGinitie, *Cercocarpus* sp.	146	Florissant, late Eocene, Colorado, USA	MacGinitie 1953; Wolfe and Schorn 1990; Manchester 2001a
*Crataeguscopeana* (Lesquereux) MacGinitie	33	Florissant, late Eocene, Colorado, USA	MacGinitie 1953; Manchester 2001a
*Crataegushendersoni* (Cockerell) MacGinitie	4	Florissant, late Eocene, Colorado, USA	MacGinitie 1953; Manchester 2001a
*Crataegusnupta* (Cockerell) MacGinitie, *Crataegus* sp.	10	Florissant, late Eocene, Colorado, USA	MacGinitie 1953; Manchester 2001a
*Crataegus* sp., aff. *Crataegus* spp. RP32, RP42	6	Republic, early Eocene, Washington, USA	Wolfe and Wehr 1987; Wilf et al. 2005b‡
*Holodiscuslisii* Schorn	2	Florissant, late Eocene, Colorado, USA	Schorn 1998; Manchester 2001a
*Malusflorissantensis* (Cockerell) MacGinitie	2	Florissant, late Eocene, Colorado, USA	MacGinitie 1953; Manchester 2001a
*Maluspseudocredneria* (Cockerell) MacGinitie	1	Florissant, late Eocene, Colorado, USA	MacGinitie 1953; Manchester 2001a
*Photiniapageae* Wolfe & Wehr	3	Republic, early Eocene, Washington, USA	Wolfe and Wehr 1987; Wilf et al. 2005b‡
*Prunusgracilis* (Lesquereux) MacGinitie	4	Florissant, late Eocene, Colorado, USA	MacGinitie 1953; Manchester 2001a
*Prunus* sp. RP54	2	Republic, early Eocene, Washington, USA	Wolfe and Wehr 1987; Wilf et al. 2005b‡ DeVore and Pigg 2007
*Rosa hilliae* Lesquereux, *Rosa* sp.	23	Florissant, late Eocene, Colorado, USA	MacGinitie 1953; Manchester 2001a
Rosaceae sp. RP61	2	Republic, early Eocene, Washington, USA	Wilf et al. 2005b
*Rubuscoloradensis* (MacGinitie) Wolfe & Tanai	2	Florissant, late Eocene, Colorado, USA	Wolfe and Tanai 1987
*Spiraea* sp. RP29	8	Republic, early Eocene, Washington, USA	Wolfe and Wehr 1987; Wilf et al. 2005b‡; DeVore and Pigg 2007
*Vauqueliniacoloradensis* (Knowlton) MacGinitie	52	Florissant, late Eocene, Colorado, USA	MacGinitie 1953; Manchester 2001a
*Vauquelinialineara* MacGinitie, *Vauquelinia* sp.	7	Florissant, late Eocene, Colorado, USA	MacGinitie 1953; Manchester 2001a
**Salicaceae**			
*Populuscinnamomoides* (Lesquereux) MacGinitie	2	Little Mountain, early Eocene, Wyoming, USA	MacGinitie 1969; Wilf 2000‡; Manchester et al. 2006
*Populuscrassa* (Lesquereux) Cockerell, *Populus* sp.	93	Florissant, late Eocene, Colorado, USA	MacGinitie 1953; Manchester 2001a
*Populustidwellii* Manchester et al.	3	Bonanza, middle Eocene, Utah, USA	MacGinitie 1969; Wilf et al. 2001‡; Manchester et al. 2006
*Populuswilmattae* Cockerell	4	Bonanza, middle Eocene, Utah, USA	MacGinitie 1969; Wilf et al. 2001‡
*Populuswyomingiana* (E.W. Berry) MacGinitie	1	Wasatch Fm., several sites, early Eocene, Wyoming, USA	MacGinitie 1974; Wilf 2000‡
*Salixcockerelli* Brown	8	Bonanza, middle Eocene, Utah, USA	MacGinitie 1969; Wilf et al. 2001‡
*Salixramaleyi* Cockerell, *Salix* sp.	15	Florissant, late Eocene, Colorado, USA	MacGinitie 1953; J.A. Wolfe pers. comm. in Manchester 2001a
*Salixtaxifoliodes* MacGinitie	2	Florissant, late Eocene, Colorado, USA	MacGinitie 1953
**Sapindaceae**			
*Acerflorissantii* Kirchner, *Acer* sp.	36	Florissant, late Eocene, Colorado, USA	MacGinitie 1953; Wolfe and Tanai 1987
*Aesculushickeyi* Manchester	3	Fort Union Fm., several sites, late Paleocene, Wyoming, USA	Wilf 2000‡; Manchester 2001b; Wilf et al. 2006‡
*Allophylusflexifolia* (Lesquereux) MacGinitie	10	Little Mountain & Bonanza, early and middle Eocene, Wyoming & Utah, USA	MacGinitie 1969; Wilf 2000‡; Wilf et al. 2001‡
*Athyanahaydenii* (Lesquereux0 MacGinitie	106	Florissant, late Eocene, Colorado, USA	MacGinitie 1953
*Bohleniainsignis* (Lesquereux) Wolfe & Wehr	16	Florissant, late Eocene, Colorado, USA	Wolfe and Wehr 1987; Manchester 2001a; McClain and Manchester 2001
“*Cardiospermum*” *coloradensis* (Knowlton) MacGinitie	5	Little Mountain & Bonanza, early and middle Eocene, Wyoming & Utah, USA	MacGinitie 1969; Wilf 2000‡; Wilf et al. 2001‡; Jud et al. 2021
“*Cardiospermum*” *terminalis* (Lesquereux) MacGinitie, “*Cardiospermum*” sp.	41	Florissant, late Eocene, Colorado, USA	MacGinitie 1953; Manchester 2001a; Jud et al. 2021
*Koelreuteriaallenii* (Lesquereux) Edwards	28	Florissant, late Eocene, Colorado, USA	MacGinitie 1953; Manchester 2001a; Wang et al. 2013
Sapindaceae sp. TY018	18	Laguna del Hunco, early Eocene, Chubut, Argentina	Wilf et al. 2005a
**Schoepfiaceae**			
*Schoepfiarepublicensis* (La Motte) Wolfe & Wehr	2	Republic, Wasatch Fm., early Eocene, Washington & Wyoming, USA	Wolfe and Wehr 1987; Wilf 2000‡; Wilf et al. 2005b‡
**Theaceae**			
Theaceae sp. RP49	2	Republic, early Eocene, Washington, USA	Wolfe and Wehr 1987; Wilf et al. 2005b‡
**Trochodendraceae**			
*Trochodendronnastae* Pigg et al.	1	Republic, early Eocene, Washington, USA	Pigg et al. 2001; Wilf et al. 2005b‡ Manchester et al. 2018
*Ziziphoidesflabellum* (Newberry) Crane et al.	9	Fort Union Fm., several sites, early Paleocene, Montana, North Dakota, & Wyoming, USA	Crane et al. 1991; Johnson 2002‡; Wilf et al. 2006‡
*Ziziphoides* sp. RP37	1	Republic, early Eocene, Washington, USA	Pigg et al. 2001; Wilf et al. 2005b‡
**Ulmaceae**			
*Cedrelospermumlineatum* (Lesquereux) Manchester, *Cedrelospermum* sp.	978	Florissant, late Eocene, Colorado, USA	Manchester 1989a, 2001a
*Cedrelospermumnervosum* (Newberry) Manchester	30	Little Mountain & Bonanza, early and middle Eocene, Wyoming & Utah, USA	Manchester 1989a; Wilf 2000‡; Wilf et al. 2001‡
*Ulmitesmicrophylla* (Newberry) Manchester	1	Wasatch Fm. several sites, early Eocene, Wyoming, USA	Wilf 2000‡; Manchester 2014
*Ulmus* sp. RP17, *Zelkova* sp. RP50	8	Republic, early Eocene, Washington, USA	Wolfe and Wehr 1987; Wilf et al. 2005b‡ Denk and Dillhoff 2005
*Ulmustenuinervis* Lesquereux, Ulmaceae sp., *Ulmus* sp.	30	Florissant, late Eocene, Colorado, USA	Manchester 1989b, 2001a
**Vitaceae**			
“*Vitisflorissantella*”	11	Florissant, late Eocene, Colorado, USA	MacGinitie 1953, 1969; Manchester 2001a

† Fossil sites listed are only those that sourced the fossils in this dataset and may not include type localities and other occurrences of the species. ‡ Occurrence reference for the dataset in this paper, when different from the taxonomic reference. § Doweld (2016) proposed revision of *Celtisaspera* to *Rhamnitesasperus* (Newberry) Doweld, which we acknowledge but do not adopt here.
